# Somersault of *Paramecium* in extremely confined environments

**DOI:** 10.1038/srep13148

**Published:** 2015-08-19

**Authors:** Saikat Jana, Aja Eddins, Corrie Spoon, Sunghwan Jung

**Affiliations:** 1 Department of Engineering Science and Mechanics, Virginia Tech, Blacksburg, VA 24061, USA

## Abstract

We investigate various swimming modes of *Paramecium* in geometric confinements and a non-swimming self-bending behavior like a somersault, which is quite different from the previously reported behaviors. We observe that *Paramecia* execute directional sinusoidal trajectories in thick fluid films, whereas *Paramecia* meander around a localized region and execute frequent turns due to collisions with adjacent walls in thin fluid films. When *Paramecia* are further constrained in rectangular channels narrower than the length of the cell body, a fraction of meandering *Paramecia* buckle their body by pushing on the channel walls. The bucking (self-bending) of the cell body allows the *Paramecium* to reorient its anterior end and explore a completely new direction in extremely confined spaces. Using force deflection method, we quantify the Young’s modulus of the cell and estimate the swimming and bending powers exerted by *Paramecium*. The analysis shows that *Paramecia* can utilize a fraction of its swimming power to execute the self-bending maneuver within the confined channel and no extra power may be required for this new kind of self-bending behavior. This investigation sheds light on how micro-organisms can use the flexibility of the body to actively navigate within confined spaces.

*Paramecia* are amongst the most ubiquitous ciliary microorganisms in nature, and their various species are often found to inhabit ponds, lakes and marine water bodies^1^,^2^. They have been reported to swim with speeds of a few millimeters per second^3^ and have also been used as indicators in bioassays to detect bacteria level in soils^4^ or the concentration of heavy metals in sludge^5^. The nature of the ciliary beat around *Paramecium* and the kinematics of the helical swimming pattern has continued to inspire experimentalists^6^,^7^,^8^ and theorists^9^,^10^,^11^,^12^ over the past few decades. The natural habitats of such microorganisms often consist of decayed matter, soil, debris and extremely confined spaces. During *Paramecium’s* navigation in the complicated natural environment, *Paramecium* might come into contact with crevices, obstacles or bio-flocs; possibly of shapes and sizes similar to its size^13^,^14^. While chemotaxis^15^, gravikinesis^16^, galvanotaxis^17^ and swimming characteristics in the bulk of fluid^18^ for *Paramecium* is well characterized, its swimming behavior in confined spaces/geometry remains relatively unexplored^19^.

Confined spaces or boundaries often bring about many surprising characteristics in a variety of swimmers, in quite unexpected and different ways. For example, bacteria and spermatozoa exhibit accumulation near flat surfaces^20^,^21^ or show circular swimming tracks due to hydrodynamic effects^22^,^23^. A suspension of spermatozoa, when injected into micro-fluidic geometries show preferential swimming along surfaces^24^. The length of the cilia/flagella has been found to play an important role in governing the scattering angle of spermatozoa and chalmydomonas after collision with a wall^25^. A bacteria after running into a wall can reorient and exhibit long residence times on surface^26^ or reverse its direction by reorienting the flagella, thereby making entry and exit swimming tracks indistinguishable^27^. A helically swimming *Paramecium* on collision with a wall exhibits avoidance behavior; during which it slightly moves backwards, gyrates its body and finally resumes its directional swimming^6^.

The flexibility of a swimmer coupled with boundaries and/or external cues can trigger an active change in the cell shape or result in a change of the swimming mode. Bacteria in sub-micron constrictions^28^ and fabricated micro-structures^29^ show adaptation to confinements by growing and/or dividing into anomalous shapes. Growing yeast cells when placed in small chambers tend to buckle and exhibit bent shapes^30^ and monotrichous bacteria have been found to utilize buckling of flagellar hook to execute sharp turns^31^. Larger organisms like fish swim by undulating their body^32^ and can also execute a C-shaped bending of the body by using muscles in order to abruptly change the swimming direction^33^. In micro-world, prey change the radii and pitch of their swimming helix to escape the predators^34^, and *Paramecium* shoot out trichocysts to exhibit evasive maneuvers in response to a threat^35^,^36^.

In this paper, we explore transitions in swimming responses of *Paramecium* in confined spaces, and report an interesting feature of the cell buckling in response to extreme confinements as seen in Fig. 1. *Paramecia* that are initially placed in quasi-infinite fluids, and are found to mostly execute directional helices (ballistic swimming) as they explore the free space. When *Paramecia* are further confined to thin fluid films, many swimmers transition from ballistic swimming to meandering and exhibit a large number of abrupt turns. In rectangular channels, a meandering *Paramecium* can push on the confining walls and execute self bending of the body to reorient in a completely new direction. We use a force deflection method to measure the elasticity of the cell body and investigate the swimming and bending power of the cell during the different maneuvers.

## Results

### Different swimming behaviors in quasi-infinite fluid

**Figure 2:**
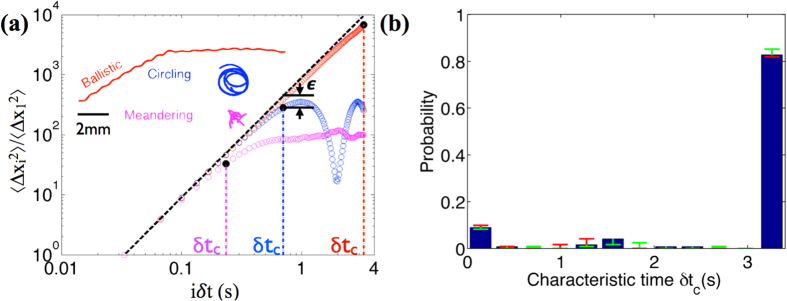
Analysis of swimming tracks and probability distribution of characteristic times for swimmers in semi-infinite fluid medium. (**a**) Plot of normalized mean square displacements vs. time difference of three different swimming trajectories as they swim in the large drop of fluid: Ballistic, meandering and circling. Only the first 100 frames of the recorded tracks are used for analysis. (Color codes are same in inset and figure). The black dashed line represents the *δt*^2^ line corresponding to ideal ballistic motion MSD_*ideal*_. Black dots represent the times at which significant deviation occurs from the ideal ballistic motion (*ε* = 20%). (**b**) Probability distribution of characteristic times *δt*_*c*_ in 2D semi-infinite fluid medium for *ε* = 20%. The red and green bar shows the changes in the characteristic time distribution when *ε* = 15% or 25% is considered.

To understand the swimming characteristics of *Paramecium*, we undertake an analysis of the recorded tracks. *Paramecia* swimming in quasi-infinite fluid media (a large drop on a glass slide) and are observed to execute three different types of trajectories as shown in Fig. 2. The first is the ballistic motion; in which *Paramecium* swims along a sinusoidal path and shows a large displacement from its starting point (shown as red curves in Fig. 2(a)). In the second case, *Paramecia* circles around and comes very close to its starting point at different times (shown as blue curves in Fig. 2(a)). The third type is a meandering mode in which *Paramecium* tends to move around locally without any large displacements from its original position (shown as purple curves in Fig. 2(a)).

To differentiate the various swimming behaviors, the mean square displacement is used as a primary measure. As a reference, we define an idealized situation in which *Paramecium* swims with constant velocity along a straight line without any turning motion. Then ideal ballistic motion has the mean square displacement (*MSD*_*ideal*_ = 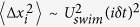 where *U*_*swim*_ is the swimming speed) and would exhibit a slope of 2 on the log-log plot of mean squared displacement vs. *δt*. For analysis, all our experimentally recorded tracks are truncated to 100 frames, so that the total time *T* of individual tracks is 3.3 s (the time interval *δt* is (1/30) s). Truncating the movies to 100 frames allows us to minimize the errors in the Matlab tracking program arising due to intersecting *Paramecium* tracks and also helps us to ensure that the full trajectories of ballistic swimmers are captured within the field of view. Subsequently, the mean squared displacements 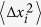 of recorded tracks are calculated as 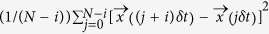 where *i*, *j* are integers. We then compare the normalized mean squared displacement (MSD) of experimentally recorded trajectories with the case of idealized ballistic swimming which is denoted by the black dotted line as in Fig. 2(a). For the case of sinusoidal path, the plot of (*MSD*_*ballistic*_) is almost a straight line which closely follows the black dotted line showing large displacements for short as well as long timescales. In the case of circling motion, the *MSD* shows dips at certain time intervals since the *Paramecium* comes very close to its original position. It is worth noting that this circling motion is rarely observed, so we will neglect this swimming behavior in the later analysis. The meandering motion shows ballistic characteristics at very short timescales, however for longer timescales the *MSD*_*meandering*_ drops off abruptly when compared to ballistic swimming, indicating a loss of memory of an initial direction of the swimmer.

To distinguish between the sinusoidal ballistic and the meandering swimmers, we calculate the absolute deviation of the mean squared displacement 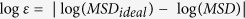 for the trajectories, and find the timescale when the *MSD* deviates 20% from the ideal ballistic case (*ε* = 1.2). The time when this deviation starts is termed as “Characteristic time” (*δt*_*c*_) and is an indicator that the motion has significantly deviated from the ideal ballistic swimming. By extracting this timescale signature from recorded tracks (a total of 3103 swimming tracks in different configurations), we get a probability distribution of the characteristic times by counting trajectories falling into a specific time interval. The probability distribution plots in different film thicknesses allow us to characterize swimming behavior executed by the swimmers in different configurations. For quasi-infinite fluid the probability distribution plot of the characteristic time (*δt*_*c*_) shows a peak at 3.3 s (as shown in Fig. 2(b)), indicating that most of the swimmers have ballistic like characteristics as they swim in semi-infinite fluid. In this study, we classify the swimmers as ballistic (*δt*_*c*_ > 3.0 s; 90% of the total swimming duration) or meandering (*δt*_*c*_ < 3.0 s) based on the difference in characteristic time.

### Ballistic to meandering transition in thick and thin fluid films

**Figure 3:**
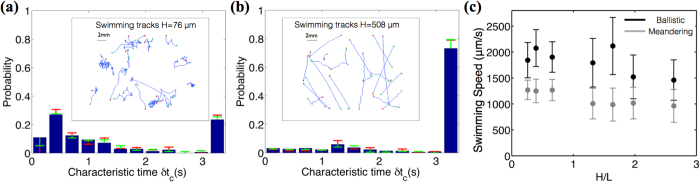
Probability distribution of characteristic times for *Paramecia* swimming in fluid films of different thicknesses. (a) Probability distribution of characteristic times in 76 μm thin film, showing high peaks at smaller timescales indicating meandering motions. (b) Probability distribution of characteristic times in 508 μm thin film, showing one single peak at a larger timescale indicating dominating ballistic motions. Insets show the visuals of recorded tracks. (**c**) Swimming speeds of ballistic and meandering *Paramecia* in different thickness fluid films. Error bars represent one half standard deviation values.

*Paramecia* are placed to swim in fluid films of varying thickness, and ballistic and meandering swimmers are distinguished using the method described in the previous section. The probability distribution of characteristic times in thick fluid films (as shown in Fig. 3(a); *H* = 508 *μ*m) has the highest peak in the bin of 3.0 s < *δt*_*c*_ < 3.3 s; showing the trend that most of swimmers display sinusoidal ballistic characteristics. But, what happens if we decrease the thickness of the fluid films in which *Paramecia* are swimming ? Inset of Fig. 3(b) shows the tracks of *Paramecium* in the thin film (*H* = 76 *μ*m); which predominantly shows meandering motion with a large abrupt number of turns.

In the case of *Chlamydomonas*, spermatozoa or bacteria the collision with a wall might result in a simple scattering effect or may show long residence times near the boundary. However, in case of *Paramecium*, the collision with a wall triggers ‘avoidance behavior’, during which the *Paramecium* backs up and gyrates its body about a mean position. In thin films that have thickness similar to the width of the cell body, the swimming space is severely constrained. This leads to a series of successive collisions with the wall and causes the *Paramecium* to move back and forth exhibiting meandering behavior with a large number of turns. Since, such a behavior causes very little displacements and frequent reorientations, the characteristic time for such swimmers tend to be on the lower side. This is confirmed by the probability distribution of characteristic time for the thin film (*H* = 76 *μ*m) that shows a new peak for 0.3 s < *δt*_*c*_ < 0.6 s in addition to the ballistic-motion peak (in the bin of 3.0 s < *δt*_*c*_ <  3.3 s); indicating that a large fraction of swimmers switch from ballistic to meandering swimming. The slightly elevated measurements of swimming speeds in thin fluid films as seen in Fig. 3(c) would be an artifact due to the fact that the swimming speeds are measured in the 2D projected plane, which neglects the vertical component and therefore systematically underestimates the swimming speed in larger gap thicknesses.

**Figure 4:**
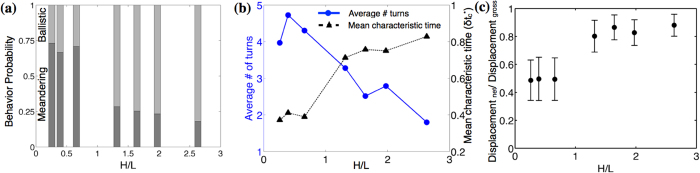
Characteristics of the swimmers in films of varying thickness. (**a**) Probability of meandering and ballistic swimming depending on the gap thickness. (**b**) Average number of turns executed by the swimmers and the mean characteristic time in thick and thin fluid films. (**c**) Ratio of net to gross displacements. The error bars indicate a half standard deviation of data. This ratio measures the tortuosity of the swimming tracks; lower values indicate higher tortuosity.

For *H* = 508 *μ*m, 78% of the swimmers are ballistic whereas in 76 *μ*m-thick films only 27% of *Paramecia* swim ballistically indicating a transition from ballistic to meandering mode in smaller gaps as seen in Fig. 4(a). The mean characteristic time (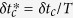) which can be thought of as a measure of directional persistence of the cell also shows an increasing trend in Fig. 4(b); confirming that in small gap thickness the orientational memory is lost at smaller times. We also measure the total number of turns for the tracks; defined by the angle included (greater than 45°) by tangents between the successive recorded positions and find that the number of turns executed in thinner films is about 2.5 times more as compared to the thicker ones (Fig. 4(b)). A measure of the tortuosity/straightness of the path given by the ratio of displacement between the first and last frame to the total distance travelled by organism^37^ shown in Fig. 4(c); also demonstrates the fact that in smaller gaps the trajectories executed are more jagged.

### Meandering to bending transition in rectangular channels

**Figure 5:**
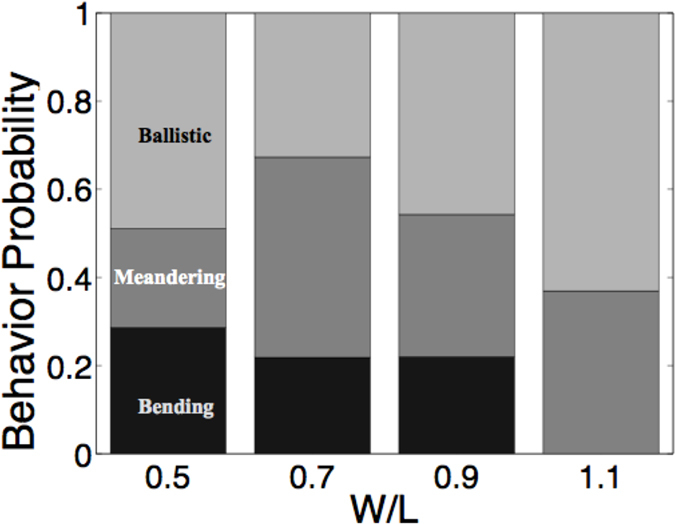
Probabilities of different swimming modes within channels. Probability of ballistic swimming, meandering, or bending in PDMS channels with different level of confinements. (*L* denotes the length of the individual *Paramecium* and *W* denotes the width of the channel).

We further studied the various behviors exhibited by *Paramecium* in Poly-dimethyl siloxane (PDMS) channels of height (*H* = 80 *μ*m) and varying widths (*W* = 120, 140, 150, 160 and 180 *μ*m) as described in the Methods section. Within these quasi-1D channels, ballistic motion as well as meandering motion are observed, however, in certain cases *Paramecia* are observed to buckle their body and abruptly change their swimming direction. The phenomenon occurs due to the confinement effect which constrains *Paramecia* to touch and exert forces on the walls. A probability plot in Fig. 5 shows that in the optimal range of 0.4 < *W*/*L* < 1.0 values for which bending events are more probable. For larger channel widths (*W*/*L* > 1), *Paramecium* cannot touch both walls simultaneously and therefore cannot bend itself.

The self-bending allows the cell to change its locomotion direction in extremely confined environments when meandering motion would not suffice. The bending of the cell which has not been reported previously, may be crucial while the cell navigates extremely small spaces within its natural environments. Experimental observations of the self-bending phenomena in *Paramecia* show that the cell initially touches and slides along both the walls. The posterior end anchors onto the wall and remains fixed; while the anterior end keeps on sliding along the other wall causing the cell body to buckle like a bent bow. This sliding motion is presumably connected to the swimming force that *Paramecium* uses in wider channels. Once the body reaches the maximum curvature, then the continuous motion of the anterior end relaxes the bent cell back to its original shape. Based on the above description of the self-bending, we hypothesize that the cell expends some of its swimming power by exerting forces on the wall to bend itself.

### Measurement of Young’s Modulus

**Figure 6:**
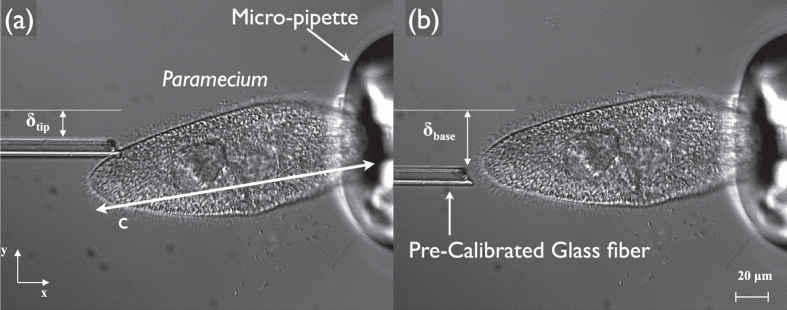
Force deflection technique to measure the elasticity of *Paramemcium*. (**a**) Deflected state of *Paramecium* and the displacement of the glass fiber from the reference position. (**b**) Undeflected state of the fiber after being retracted horizontally and the *Paramecium* has also returned to its original undeflected state.

To estimate the power spent during self-bending, the stiffness of the cell body needs to be determined. We use force deflection technique^38^ to quantify the Young’s modulus of the immobilized cell. A glass micro-fiber manufactured by extrusion is calibrated by hanging weights and is used to deflect the free end of the *Paramecium*. The *Paramecium* was held by suction using a glass pipette, and the flexible glass micro-fiber of known stiffness (*k*) was placed against the free end. Then the base of the glass fiber (out of view) was displaced forcing the *Paramecium* to deflect a distance (*δ*_*tip*_) from it’s equilibrium position as shown in Fig. 6(a).

The glass fiber was the horizontally retracted, and the fiber was straightened which allowed for measurement of the fiber’s base displacement (*δ*_*base*_). Force (*F*_*fiber*_) applied to the *Paramecium* due to displacement was determined by *F*_*fiber*_ = *k*(*δ*_*base*_−*δ*_*tip*_). By knowing the value of the force exerted (about 20 ~ 60 nN for different trials) we can finally calculate the elastic modulus of the cell as *E* = *F*_*fiber*_*c*^3^/3*Iδ*_*tip*_ where *c* denotes the length of the cell and *I* donates the moment of inertia of the cell body. Three different cells and multiple (15 ~ 17 force deflection trials) allowed us to find the elastic modulus to be in range of 4.1 ± 0.4 kPa^39^; which is significantly lower than the cell wall of prokaryotic bacteria *E. Coli*^40^,^41^ and also the cilium^42^.

### Power exerted by *Paramecium* during bending in quasi 1D channel

**Figure 7:**
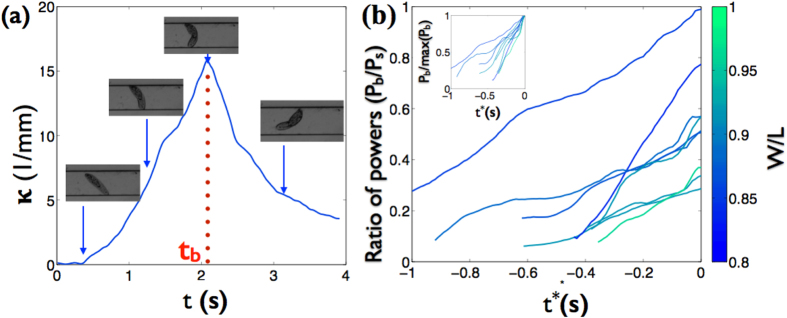
Bent states of *Paramecium* within channels. (**a**) Plot of the curvature of a *Paramecium* while bending at different instances (t). The time at which maximum curvature occurs is denoted by t_*b*_. (**b**) Ratio of bending to swimming power for *Paramecia* vs t^*^ = *t* − *t*_*b*_. This graph shows that the bending power is always less than the swimming power. The upper inset shows the normalized bending powers (*P*_*b*_/max(*P*_*b*_)) vs t^*^ for the different cases.

As *Paramecium* maneuvers to bend within the channel; the posterior end gets anchored to one of the walls. The time to anchor varies across individual organisms and is influenced by factors like roughness of the walls and local adhesion between cilia and the wall. During the anchoring period, *Paramecium* has very little curvature as seen in Fig. 7(a) (*t* < 0.5 s). The *Paramecium* then progressively deforms its body and at the point of maximum curvature assumes a bow-like shape during 0.5 s < *t* < 2.2 s in Fig. 7(a). The maximum body curvature is followed by a relaxation to the unbent state after which the *Paramecium* either resumes swimming in the opposite direction, or prepares for another bending event.

In the case of swimming *Paramecium*, the swimming power (*P*_*s*_) is estimated as *P*_*s*_ = 6*πμV*^2^*L* ~ 14 pW where *μ* ~ 10^−3^ Pa·s is the viscosity of water, *V* ~ 2000 *μ*m/s is the average ballistic swimming speed in 76 *μ*m gap thickness (see Fig. 3c) and *L* ~ 200 *μ*m is the length of the organism. The bending power (*P*_*b*_) of the cell can be approximated as *P*_*b*_ = *EILκ*∂*κ/*∂*t*, where *E* ~ 4 kPa is the Young’s modulus, *I* = *πr*^4^/4 ≈5 × 10^−12^ m^4^ is the moment of inertia based on the average width (*r* ≈20 *μ*m), *κ* is the curvature of bending *Paramecium*, and the term ∂*κ/*∂*t* denotes the variation of the curvature with time. To measure *κ*, image sequences of *Paramecium* during bending events are converted into black and white image. Then, the body centerline is extracted and is best-fitted by a curved line of constant radius (*R*). The inverse of the fitting radius yields the body curvature (*κ* = 1/R). As shown in Fig. 7(a), the curvature is monotonically increasing until the maximum is reached. After that, the *Paramecium* relaxes back to its unbent state. The bending power is measured in cases of only *W*/*L* > 0.8. We also observed the bending events when *W*/*L* < 0.8, however, the bending becomes highly nonlinear like forming a kink in the middle of the body, and falls outside the scope of our estimates using a linear theory.

Figure 7(b) shows the plot of the ratio of bending to swimming power (*P*_*b*_/*P*_*s*_) vs time t^*^(= *t* − *t*_*b*_; where *t*_*b*_ is the time when *Paramecium* reaches the maximum curvature). The bending power exerted by *Paramecia* is less than the swimming power since the power ratio never goes beyond 1. In addition, this power ratio increases as the channel width gets smaller, indicating *Paramecium* uses more power to bend its body in narrower channels. During the bending, the lateral displacement of the cell is quite small, therefore we assume swimming and bending events to be mutually exclusive. This leads us to conclude that a fraction of the power that would be otherwise spent for swimming is utilized to bend the cell as a part of swimming course within the channel, thereby allowing the cell to switch directions.

## Discussion

The experimental investigations described here reveals the complicated nature of the swimming tracks executed by *Paramecium multimicronucleatum* as it interacts with its fluidic environment. We start by verifying the conventional notion that *Paramecium* swims helically in quasi-infinite fluid media, and progressively show different swimming behaviors of *Paramecium* by introducing various confinements. When *Paramecia* are placed in thin films, we find that *Paramecia* meander around due to frequent collision and reorientation events arising from the confining boundaries. In rectangular channels the meandering ciliate anchors itself to the walls and bends its flexible body to change its swimming orientation. We measure the Young’s modulus of the cell and show that the organism can use a fraction of its swimming power to bend within the channel. The investigation sheds light on the previously unknown bending maneuver executed by *Paramecium* in confined geometries and also shows how body deformability can be exploited by slender micro-organisms to navigate and/or escape from the complicated geometries in their habitat.

## Methods

### Cell Culture

**Figure 8:**
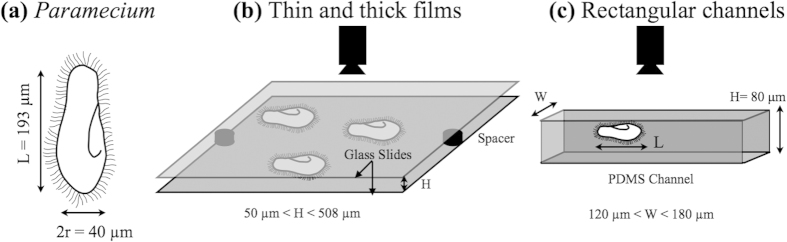
Experimental setups : (**a**) Schematic showing the dimensions of *Paramecium*. (**b**) Fluid films of varying thickness (50 < *H* < 508 *μ*m). (**c**) Rectangular channels of 80 *μ*m height with different widths (120 < *W* < 180 *μ*m).

Wild type *Paramecium Multimicronucleatum* (Part No. 131540) initially obtained from ATCC 30842 by Carolina Biological Supply were ordered for our experiments. The cells were centrifuged; washed and are cultured in a medium consisting of boiled spring water and wheat seeds. Washed cells were made to grow in a controlled temperature environment of 22 °C. The cells were isolated during their growth phase and were washed twice in Tris-HCl buffer solution consisting of 9 mM CaCl_2_, 3 mM KCl and 5 mM Tris-HCl with pH adjusted to 7.2. The cells showed renewed vigor when introduced into this solution and were allowed to equilibrate for 30 minutes prior to starting the experiments. The cells were found to be of a prolate spheroid shape with the major axis of the cell bodies measuring 193 ± 24 *μ*m and the minor axis being 40.3 ± 5.6 *μ*m (as seen in Fig.8(a)). The propelling organelles known as the cilia project out front the protective covering called pellicle and beat at a frequency of about 30 Hz^19^ to create metachronal waves. Ballistically swimming *Paramecia* are found to propel mostly along the anterior direction in a left-handed helical trajectory in the quasi-infinite fluid with the velocities measuring 2.397 ± 1.001 mm/s.

### Experimental Methods—Thin and thick films

To simulate the effect of fluid films of varying thickness we took two clean glass slides (VWR Vistavision) 76.2 × 25.4 × 1 mm. Two plastic spacers having a constant thickness (*H* = 50, 76, 127, 254, 317, 381, or 508 *μ*m) were placed at the edge of the glass slide. A controlled volume of the washed culture media was pipetted onto the slide and the other glass slide was put on top. A fiberoptic light source (Fiber-lite MI 152) was used to provide illumination from the side that allowed us to obtain dark-field images of the swimming organisms as shown in Fig. 8(b). A Sony handycam (Sony HDR-XR100) with a 4 × zoom lens was used to record 2D projected swimming tracks of multiple *Paramecia* at 30 fps.

### Experimental Methods—Rectangular channels

For quasi 1D confinements, microfluidic channels were used with a fixed height (*H* = 80 *μ*m) and varying widths (*W* = 120, 140, 150, 160 and 180 *μ*m). Mould masters ordered from Stanford Micro-fluidics foundry were cleaned with isopropyl alcohol and dried in a stream of compressed air. Sylgard 184 polymer and the curing agent were mixed in ratio of 10:1 by weight and poured over the mould master^43^. The mixture was degassed and allowed to bake at 65 °C. Finally the cured polymer was cut into pieces and bonded to glass slide using a plasma cleaner and *Paramecia* were introduced into the channels using a pipette. Swimming *Paramecia* were observed under Olympus CKX-41 microscope at 4 × magnification and their swimming tracks are recorded at 100 fps for 4 sec duration using a high-speed camera (Pixel-Link) as shown in Fig. 8(c).

### Experimental Methods—Elasticity Measurement

Micro-pipettes were used to apply suction to *Paramecia* and hold them in place for the experiments involving determination of Young’s modulus. Glass pipette tips were secured in the holder of Micro-forge (MF 900, Narishige Inc.) and heat was applied (using a micropipette puller) which caused the hollow section to be extruded under its own weight. Halfway through the length, where the diameter of the extruded section has become sufficiently low (~ 20 *μ*m); heat is applied so that the thin region bends and forms an angle with the vertical. This was done to ensure that the micro-capillary can easily be dipped into the fluid containing *Paramecia*.

While the *Paramecia* were held in place with the micro-pipette, their distal end were deflected using a glass fiber. The fibers were made from borosilicate glass rods with diameters of 0.5 mm. The rods were first extruded and then the tip was heated until a blob of glass formed at the tip of the rod. The heating coil of the Micro-forge was then brought in contact with the blob and quickly withdrawn in a perpendicular direction to extrude a thin fiber. The length of the fiber was clipped to match the stiffness of *Paramecium* and was then used to deflect the *Paramecium* held in place.

## Additional Information

**How to cite this article**: Jana, S. *et al.* Somersault of *Paramecium* in extremely confined environments. *Sci. Rep.* **5**, 13148; doi: 10.1038/srep13148 (2015).

## Figures and Tables

**Figure 1:**
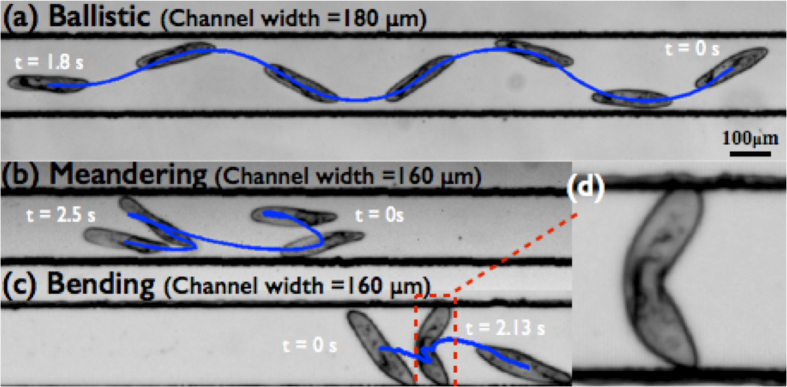
Three different kinds of swimming modes seen during locomotion of *Paramecium* in confined channels : (**a**) Ballistic helical swimming with a net direction, (**b**) meandering motion with turns, (**c**) sudden bending to change the direction of swimming, and (**d**) zoomed-in image for self-bending. (See the supplement video).

## References

[b1] Foissner W., Chao A. & Katz L. Diversity and geographic distribution of ciliates (protista: Ciliophora). In Foissner W. & Hawksworth D. (eds.) Protist Diversity and Geographical Distribution, Vol. 8 of Topics in Biodiversity and Conservation, 111–129 (Springer Netherlands, 2009).

[b2] Wichterman R. The biology of Paramecium. (Plenum Press, New York, 1986).

[b3] Machemer H. & Machemer-Rohnisch S. Is gravikinesis in *Paramecium* affected by swimming velocity? Eur. J. Protistol. 32, 90–93 (1996).

[b4] Habte M. & Alexander M. Further evidence for the regulation of bacterial populations in soil by protozoa. Arch. Microbiol. 113, 181–183 (1977). 879960 10.1007/BF00492022

[b5] Madoni P., Davoli D. & Gorbi G. Acute toxicity of lead, chromium, and other heavy metals to ciliates from activated sludge plants. B. Environ. Contam. Tox. 53, 420–425 (1994). 10.1007/BF00197235 7919720

[b6] Jennings H. Behavior of the lower organisms, vol. 10 (The Columbia university press, The Macmillan company, agents, London, 1906).

[b7] Machemer H. Ciliary activity and the origin of metachrony in *Paramecium*: effects of increased viscosity. J. Exp. Biol. 57, 239–259 (1972). 5075893 10.1242/jeb.57.1.239

[b8] Jung I., Powers T. R. & Valles J. M. Evidence for two extremes of ciliary motor response in a single swimming microorganism. Biophys. J. 106, 106–113 (2014). 24411242 10.1016/j.bpj.2013.11.3703 PMC3907369

[b9] Crenshaw H. C. Kinematics of helical motion of microorganisms capable of motion with four degrees of freedom. Biophys. J. 56, 1029–1035 (1989). 2636879 10.1016/S0006-3495(89)82748-1 PMC1280601

[b10] Crenshaw H. C. & Edelstein-Keshet L. Orientation by helical motion II. Changing the direction of the axis of motion. Bull. Math. Biol. 55, 213–230 (1993).

[b11] Crenshaw H. C. Orientation by helical motion III. Microorganisms can orient to stimuli by changing the direction of their rotational velocity. Bull. Math. Biol. 55, 231–255 (1993).

[b12] Zhu L., Lauga E. & Brandt L. Low-Reynolds-number-swimming in a capillary tube, J. Fluid Mech., 726, 285–311 (2013).

[b13] Jonsson P. & Johansson M. Swimming behaviour, patch exploitation and dispersal capacity of a marine benthic ciliate in flume flow. J. Exp. Mar. Biol. Ecol. 215, 135–153 (1997).

[b14] Darbyshire J. The use of soil biofilms for observing protozoan movement and feeding. FEMS Microbiol. Lett. 244, 329–333 (2005). 15766786 10.1016/j.femsle.2005.02.001

[b15] Kung C. & Saimi Y. The physiological basis of taxes in Paramecium. Ann. Rev. Physiol. 44, 519–534 (1982). 6280592 10.1146/annurev.ph.44.030182.002511

[b16] Guevorkian K. & Valles J. M. Swimming Paramecium in magnetically simulated enhanced, reduced, and inverted gravity environments. P. Natl. Acad. Sci. USA 103, 13051–13056 (2006). 10.1073/pnas.0601839103 PMC1559751 16916937

[b17] Itoh A. Motion control of protozoa for bio-mems. IEEE-ASME T. Mech. 5, 181–188 (2000).

[b18] Brennen C. & Winet H. Fluid mechanics of propulsion by cilia and flagella. Ann. Rev. Fluid. Mech. 9, 339–398 (1977).

[b19] Jana S., Um S. H. & Jung S. Paramecium swimming in capillary tube. Phys. Fluids. 24 041901 (2012).

[b20] Li G. *et al.* Accumulation of swimming bacteria near a solid surface. Phys. Rev. E 84, 041932 (2011). 10.1103/PhysRevE.84.041932 22181200

[b21] Smith D., Gaffney E., Blake J. & Kirkman-Brown J. Human sperm accumulation near surfaces: a simulation study. J. Fluid Mech. 621, 289–320 (2009).

[b22] Lauga E., DiLuzio W., Whitesides G. & Stone H. Swimming in circles: motion of bacteria near solid boundaries. Biophys. J. 90, 400–412 (2006). 16239332 10.1529/biophysj.105.069401 PMC1367047

[b23] Woolley D. Motility of spermatozoa at surfaces. Reproduction 126, 259–270 (2003). 12887282 10.1530/rep.0.1260259

[b24] Denissenko P., Kantsler V., Smith D. & Kirkman-Brown J. Human spermatozoa migration in microchannels reveals boundary-following navigation. P. Natl. Acad. Sci. USA 109, 8007–8010 (2012). 10.1073/pnas.1202934109 PMC3361448 22566658

[b25] Kantsler V., Dunkel J., Polin M. & Goldstein R. E. Ciliary contact interactions dominate surface scattering of swimming eukaryotes. P. Natl. Acad. Sci. USA 110, 1187–1192 (2013). 10.1073/pnas.1210548110 PMC3557090 23297240

[b26] Drescher K., Dunkel J., Cisneros L. H., Ganguly S. & Goldstein R. E. Fluid dynamics and noise in bacterial cell-cell and cell-surface scattering. P. Natl. Acad. Sci. USA 108, 10940–10945 (2011). 10.1073/pnas.1019079108 PMC3131322 21690349

[b27] Cisneros L., Dombrowski C., Goldstein R. & Kessler J. Reversal of bacterial locomotion at an obstacle. Phys. Rev. E 73, 030901 (2006). 10.1103/PhysRevE.73.030901 16605492

[b28] Mannik J., Driessen R., Galajda P., Keymer J. & Dekker C. Bacterial growth and motility in sub-micron constrictions. P. Natl. Acad. Sci. USA 106, 14861–14866 (2009). 10.1073/pnas.0907542106 PMC2729279 19706420

[b29] Takeuchi S., DiLuzio W., Weibel D. & Whitesides G. Controlling the shape of filamentous cells of Escherichia coli. Nano Lett. 5, 1819–1823 (2005). 16159230 10.1021/nl0507360 PMC2519610

[b30] Minc N., Boudaoud A. & Chang F. Mechanical forces of fission yeast growth. Curr. Biol. 19, 1096–1101 (2009). 19500986 10.1016/j.cub.2009.05.031 PMC2790036

[b31] Son K., Guasto J. S. & Stocker R. Bacteria can exploit a flagellar buckling instability to change direction. Nat. Phys. 9, 494–498 (2013).

[b32] Pedley T. & Hill S. Large-amplitude undulatory fish swimming: fluid mechanics coupled to internal mechanics. J. Exp. Biol. 202, 3431–3438 (1999). 10562526 10.1242/jeb.202.23.3431

[b33] Tytell E. & Lauder G. Hydrodynamics of the escape response in bluegill sunfish, Lepomis macrochirus. J. Exp. Biol. 211, 3359–3369 (2008). 18931309 10.1242/jeb.020917 PMC2669901

[b34] Sheng J. *et al.* Digital holographic microscopy reveals prey-induced changes in swimming behavior of predatory dinoflagellates. P. Natl. Acad. Sci. USA 104, 17512–17517 (2007). 10.1073/pnas.0704658104 PMC2077287 17959778

[b35] Hara R. & Asai H. Electrophysiological responses of Didinium nasutum to Paramecium capture and mechanical stimulation. Nature 283, 869–870 (1980).

[b36] Hamel A., Fisch C., Combettes L., Dupuis-Williams P. & Baroud C. Transitions between three swimming gaits in Paramecium escape. P. Natl. Acad. Sci. USA 108, 7290–7295 (2011). 10.1073/pnas.1016687108 PMC3088577 21464291

[b37] Codling E. A. Plank M. J. & Benhamou S. Random walk models in biology. J. R. Soc. Interface 5, 813–834 (2008). 18426776 10.1098/rsif.2008.0014 PMC2504494

[b38] Spoon C. & Grant W. Biomechanics of hair cell kinocilia: experimental measurement of kinocilium shaft stiffness and base rotational stiffness with Euler-bernoulli and Timoshenko beam analysis. J. Exp. Biol. 214, 862–870 (2011). 21307074 10.1242/jeb.051151 PMC3036549

[b39] Kuznetsova T., Starodubtseva M., Yegorenkov N., Chizhik S. & Zhdanov R. Atomic force microscopy probing of cell elasticity. Micron 38, 824–833 (2007). 17709250 10.1016/j.micron.2007.06.011

[b40] Deng Y., Sun M. & Shaevitz J. Direct measurement of cell wall stress stiffening and turgor pressure in live bacterial cells. Phys. Rev. Lett. 107, 158101 (2011). 22107320 10.1103/PhysRevLett.107.158101

[b41] Tuson H. *et al.* Measuring the stiffness of bacterial cells from growth rates in hydrogels of tunable elasticity. Mol. Microbiol. 84, 874–891 (2012). 22548341 10.1111/j.1365-2958.2012.08063.x PMC3359400

[b42] Baba S. A. Flexural rigidity and elastic constant of cilia. J. Exp. Biol. 56, 459–467 (1972). 5022843 10.1242/jeb.56.2.459

[b43] Xia Y. & Whitesides G. Soft lithography. Ann. Rev. Mater. Sci. 28, 153–184 (1998).

